# Zn^2+^-triggered self-assembly of Gonadorelin [6-D-Phe] to produce nanostructures and fibrils

**DOI:** 10.1038/s41598-018-29529-w

**Published:** 2018-07-26

**Authors:** Yordanka Yordanova, Willem Vanderlinden, Raphael Stoll, Daniel Rüdiger, Andreas Tosstorff, Wolfgang Zaremba, Gerhard Winter, Stefan Zahler, Wolfgang Friess

**Affiliations:** 1 Department of Pharmacy, Pharmaceutical Technology & Biopharmaceutics, Ludwig-Maximilians-Universitaet, Butenandtstrasse 5, 81377 Muenchen, Germany; 2 Department of Applied Physics and Centre for NanoScience, Ludwig-Maximilian-Universitaet, Amalienstrasse 54, 80799 Muenchen, Germany; 3 Department of Chemistry and Biochemistry, Biomolecular NMR Spectroscopy and RUBiospek, Ruhr-Universitaet Bochum, Universitaetsstrasse 150, 44780 Bochum, Germany; 4 Department of Pharmacy, Pharmaceutical Biology, Ludwig-Maximilians-Universitaet, Butenandtstrasse 5, 81377 Muenchen, Germany; 5 Veyx Pharma GmbH, Scientific Department, Soehreweg 6, 34639 Schwarzenborn, Germany

## Abstract

A synthetic derivative, GnRH [6-D-Phe], stable against enzymatic degradation, self-assembles and forms nanostructures and fibrils upon a pH shift in the presence of different concentrations of Zn^2+^ *in vitro*. Attenuated Total Reflection Fourier Transform Infrared spectroscopy (ATR–FTIR) revealed the existence of higher order assembly of Zn^2+^: GnRH [6-D-Phe]. Nuclear Magnetic Resonance spectroscopy (NMR) indicated a weak interaction between Zn^2+^ and GnRH [6-D-Phe]. Atomic Force Microscopy (AFM) showed the existence of GnRH [6-D-Phe] oligomers and fibrils. Molecular Dynamic (MD) simulation of the 10:1 Zn^2+^: GnRH [6-D-Phe] explored the interaction and dimerization processes. In contrast to already existing short peptide fibrils, GnRH [6-D-Phe] nanostructures and fibrils form in a Tris-buffered pH environment in a controlled manner through a temperature reduction and a pH shift. The lyophilized Zn^2+^: GnRH [6-D-Phe] assembly was tested as a platform for the sustained delivery of GnRH [6-D-Phe] and incorporated into two different oil vehicle matrices. The *in vitro* release was slow and continuous over 14 days and not influenced by the oil matrix.

## Introduction

A non-covalent complexation of peptides with metal ions controlling their release is a method, which resembles their storage in vesicles at physiological conditions. Insulin, for instance, is a prominent example for a peptide stored in the pancreas in the form of insoluble zinc complex^1^. The extended availability of insulin from an insoluble zinc complex has been long known and utilized. The development of insulin sustained release formulations for injection originated from studies of the insulin solubility in acetate and phosphate buffer^2^. The studies found the efficiency of insulin precipitation to be a function of pH and zinc salt concentration. Recently, a similar approach was adopted with other peptide compounds in order to achieve their controlled and sustained release. A non-covalently bound adduct of glucagon-like peptide-1 (GLP-1) agonist with zinc acetate could prolong its terminal half-life t_1/2_ after subcutaneous application from 2 h to 8.5 h. It further reduced the initial burst release of GLP-1 from the formulation vehicle, indicating its potential for long term release^3^. A precipitation of recombinant Hirudin (rHir) by zinc salt at neutral pH was as well shown to result in Zn^2+^-rHir suspension with a prolonged biological activity in rats^4^. The prepared suspension was then further optimized with regards to pH and zinc salt^5^. Similarly, corticotrophin (ACTH) was precipitated with zinc salt^6^. The addition of zinc induced the dimerization of human growth hormone (hGH). The Zn^2+^-hGH dimer was more stable than the monomeric hGH form^7,8^. The formed structures of Zn^2+^-hGH were later found to be fibrils of amyloidogenic nature, able to release monomers upon dilution^9^. Various studies have investigated short amyloidogenic peptides and their assemblies that mimic the amyloidosis process^10–14^. The amyloid structures have also been considered as a possible source for sustained release of small peptides, e.g. the GnRH analogue Leuprorelin (LHRH)^15^. In this connection, LHRH was complexed and precipitated with Zn^2+^, using sodium hydroxide (NaOH) solution for the pH adjustment. The lyophilizate was then incorporated into *in situ* forming implants^16^. The same technique for complexation and precipitation was applied in another study with GnRH, tyroliberin (TRH), dalarelin and ACTH for the formation of Zn^2+^-peptide aqueous suspension^17^. Comparable to hGH, LHRH exhibited a similar tendency to form β-sheet rich aggregates with amyloid-like character^15^. In both cases, peptides with the ability to form amyloids were investigated. The amyloid structures are highly organized stable aggregates, which in most cases are associated with neurodegenerative diseases^18^. However, in the studies with hGH and LHRH, the concept of amyloids was applied to prepare complexes capable of slowly releasing the active peptide.

**Figure 1:**
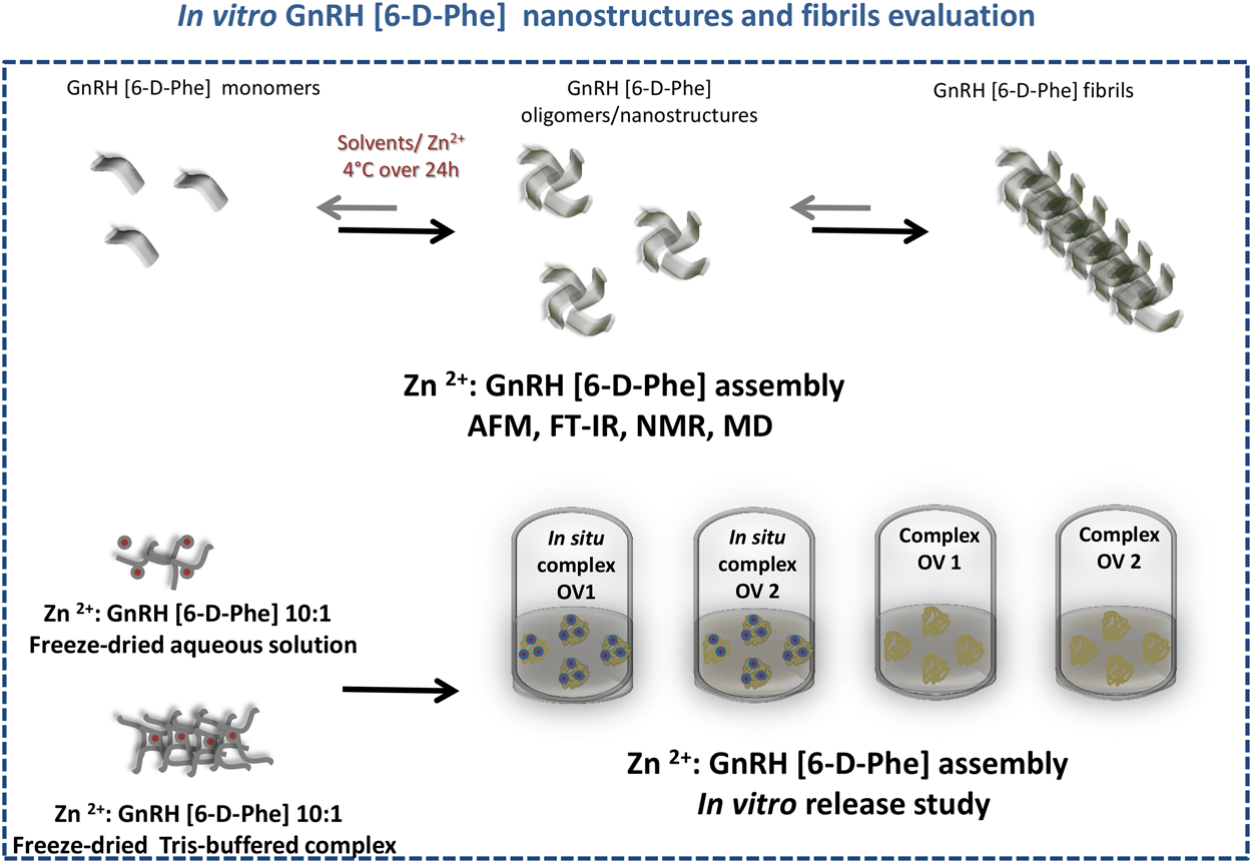
Overview of the *in vitro* GnRH [6-D-Phe] nanostructures and fibrils formation and evaluation.

Based on the “amyloid concept” from these studies, we investigated whether a controlled pH shift in the presence of different concentrations of Zn^2+^ would lead to self-assembly and formation of nanostructures and fibrils of a synthetic D-Phenylalanine (Phe) form of GnRH peptide *in vitro*. The use of the synthetic form of GnRH aimed to achieve higher stability against degradation of the assembly^19^ (Fig. 1).

## Results and Discussion

### New approach to fibrillize and self-assemble GnRH [6-D-Phe] by Zn^2+^ and controlled pH shift

In the current study we applied two techniques for fibrillizing GnRH [6-D-Phe] and compared them to each other in order to select the most suitable one. The percentage of free GnRH [6-D-Phe] was analyzed by Reversed-Phase Chromatography (RP-HPLC).

**Figure 2:**
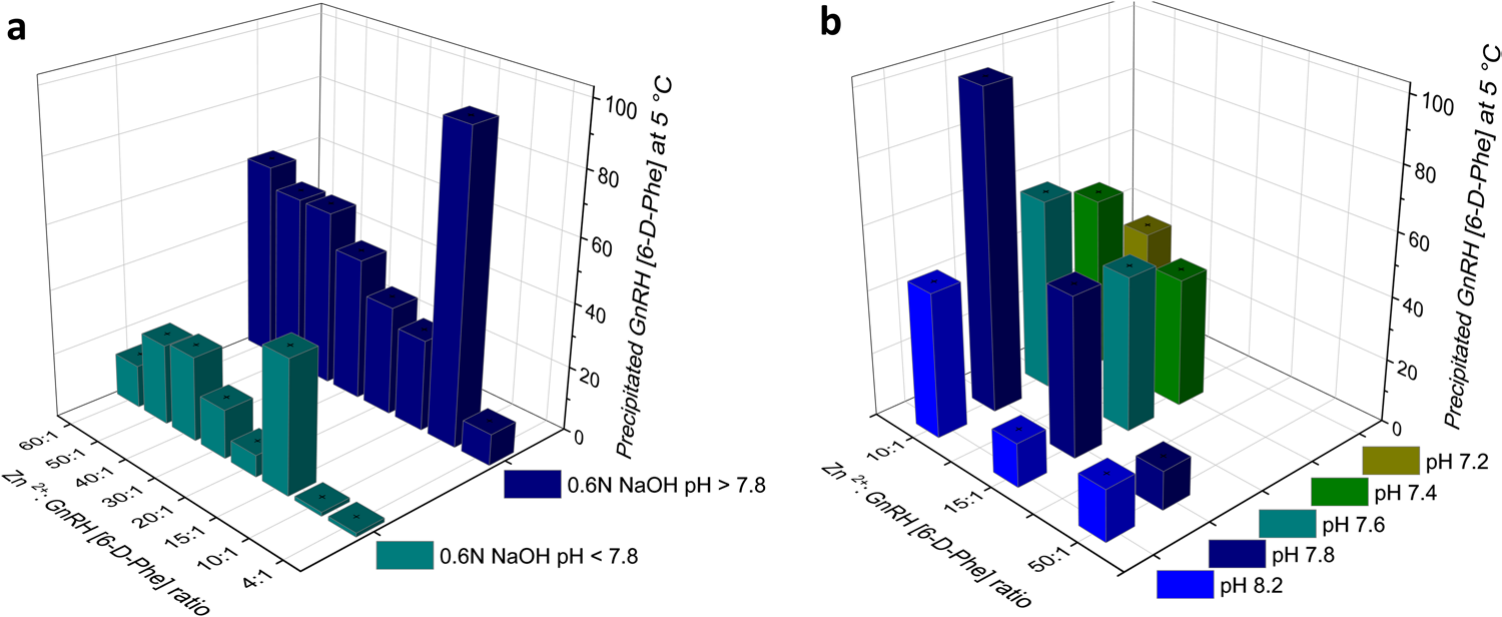
Precipitated GnRH [6-D-Phe] from Zn^2+^: GnRH [6-D-Phe] solution. (**a**) With 0.6 N NaOH at pH > 7.8 and pH < 7.8 (**b**) in 0.05 M Tris buffer at 5 °C (starting pH at 25 °C given); the left panel illustrates an increased, but uncontrolled, precipitation at molar ratio Zn^2+^: GnRH [6-D-Phe] 10:1 at pH > 7.8 with 0.6 N NaOH; the right panel illustrates a new method of a temperature controlled Tris buffer precipitation of Zn^2+^: GnRH [6-D-Phe] 10:1 at 5 °C.

The first technique was the method of choice used in earlier studies of precipitating LHRH, rHir, ACTH, TRH etc. and involved increasing the pH of the peptide solution containing Zn^2+^ from 7.2 to 8.2 by the addition of NaOH^5,16,17,20^. The GnRH [6-D-Phe] was complexed at 2.5 mg/mL from 5 mg/mL bulk concentration. Below pH 7.8 the percentage of precipitated GnRH [6-D-Phe] remained low at all Zn^2+^: GnRH [6-D-Phe] molar ratios and no correlation between molar ratio and pH could be obtained. Above pH 7.8, we observed an increase and a maximum of precipitated GnRH [6-D-Phe] of 95.9 ± 0.1% at a 10:1 Zn^2+^: GnRH [6-D-Phe] molar ratio (Fig. 2a; Supplementary Data and Information Table 1). The high percentage of precipitated peptide might be due to the interaction of the histidine (His) side chain with Zn^2+^ through its imidazole group. One major drawback of the technique is the unsatisfactory accuracy using the pH-adjustment with NaOH solution due to lack of buffer capacity at the pH value of interest. Thus upon addition of NaOH extremely unstable and high local pH values may result. This can lead on the one hand to the coinciding precipitation of Zn (OH)_2_ from a mixture of complexed, free peptide and unbound Zn^2+^ and on the other to epimerization at the serine (Ser) of GnRH [6-D-Phe], preceding a Ser hydroxyl group triggered peptide bond hydrolysis^21^.

The second technique was based on a recent study of Valery *et al*., which showed that a change of pH value and thus deprotonation of the His residue in the GnRH agonist, TRH, could trigger a self-assembly of the peptide into nanotubes^22^. This pH dependent switch can be applied in the bottom-up development of pH-responsive nanomaterials as well as for the *in vitro* induced conformational change and fibrillation of GnRH [6-D-Phe]. In our study, this was achieved through a temperature controlled pH-shift between 7.2 and 8.2 with a Tris-buffered system, as an alternative to NaOH^23^.

The percentage of precipitated peptide reached 99.2 ± 0.2% at a 10:1 Zn^2+^: GnRH [6-D-Phe] ratio in 0.05 M Tris buffer pH 7.8 after temperature reduction from 25 °C to 5 °C, over 24 h. In contrast, the precipitation efficiency in a 0.05 M Tris buffer pH 7.2 reached only 36.2 ± 0.1% (Fig. 2b). This could be explained by the measured-temperature-induced pH increase of the Tris-buffered system with 0.03 pH units per 1 °C^24^. Cooling the Zn^2+^: GnRH [6-D-Phe] in Tris buffer (pH 7.8) from 25 °C to 5 °C promoted a pH increase from pH 7.3 to pH 7.9. At this pH the His-side chain is in a deprotonated state and can form hydrogen bonds or enhance the peptide interaction with Zn^2+^ and the formation of an assembly. In contrast the solution of Zn^2+^: GnRH [6-D-Phe] in Tris buffer with pH 7.2 reached pH 7.3 at 5 °C (Supplementary Data and Information Table 2).

### Existence of higher-order assembly of GnRH [6-D-Phe]

**Figure 3:**
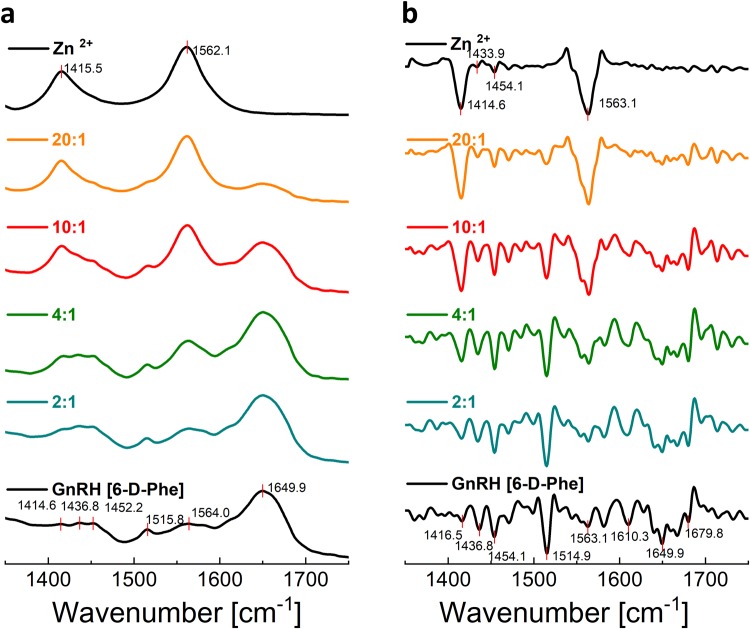
FT-IR Spectra of the Zn^2+^: GnRH [6-D-Phe] assemblies in comparison to GnRH [6-D-Phe]. (**a**) normalized, absorbance spectra (**b**) normalized second derivative of the absorbance spectra.

In order to study the conformational changes and secondary structures, resulting upon adding different molar concentrations of Zn^2+^ to GnRH [6-D-Phe], we used Attenuated Total Reflection Fourier Transform Infrared spectroscopy (ATR–FTIR). The bands at 1416 cm^−1^ and 1434–1454 cm^−1^ indicated an increase in the C-H bending and -C = O stretching vibration, respectively^25–27^ (Fig. 3a). Absorbance spectra analysis in the amide II range (1500–1600 cm^−1^) yielded two major peaks at ~1520 cm^−1^ and 1560 cm^−1^. The peak at 1560 cm^−1^ can be assigned to the N-H bend in plane and stretch. The peak at 1520 cm^−1^ has been associated in earlier studies with vibrations of heterocyclic compounds, e.g. furan ring, phenyl ring, imidazole ring and can be attributed to the stretching vibration of the –C=N bond in the imidazole heterocycle of the His side chain^28–30^ (Fig. 3a). The amide I region (1600–1700 cm^−1^) presented one major band at 1650 cm^−1^ as an indicator of an alpha-helical structure. The characteristic beta-sheet regions of 1610–1630 cm^−1^ and 1680–1700 cm^−1^ were visibly overlapped from the major alpha-helical peak. A deconvolution of the absorbance spectra could unveil a beta-sheet peak in the 1604–1617 cm^−1^ range (Supplementary Data and Information Fig. 2). With increasing Zn^2+^ molar concentrations, most stunning appears to be the change in the percentage of beta-sheet and carbonyl stretching area. The observed changes are good indicators of the initiation of oligomer formation at 4:1 and the maximum stretching of the carbonyl group at 10:1 Zn^2+^: GnRH [6-D-Phe] molar ratios (Fig. 3a, Table 1). With higher Zn^2+^ concentrations the beta-sheet peak disappeared and an increase of the carbonyl stretching due to the formation of ZnO/Zn (OH)_2_ resulted. Its strong peak at 1560 cm^−1^ coincides with the peptide amide II region. Analysis of the second-derivative amide I region showed bands at ~1611 cm^−1^ and at ~1690 cm^−1^, typical for oligomers in solution^31–36^ (Fig. 3b). This supports the hypothesis that the oligomeric structure of GnRH [6-D-Phe] forms with increasing molar concentration of Zn^2+^.

**Table 1:** Main peaks and integrated peak area in the deconvoluted normalized absorbance spectra of Zn^2+^: GnRH [6-D-Phe] assemblies in comparison to GnRH [6-D-Phe].

	Main peaks cm^−1^ (% Area)				
	-C-H bending and C=O stretching	-C=N (Imidazole)	Amide II	Beta-sheet	Amide I
GnRH [6-D-Phe]	1416 (2%) 1434 (2%) 1454 (6%)	1514 (3%)	1568 (27%)	1612 (6%)	1652 (54%)
Zn^2+^: GnRH [6-D-Phe] 2_1	1414 (4%) 1435 (5%) 1456 (7%)	1514 (2%)	1563 (17%)	1608 (10%)	1653 (54%)
Zn^2+^: GnRH [6-D-Phe] 4_1	1417 (8%) 1435 (1%) 1452 (8%)	1515 (3%)	1560 (20%)	1617 (20%)	1657 (40%)
Zn^2+^: GnRH [6-D-Phe] 10_1	1416 (13%) 1435 (1%) 1450 (7%)	1517 (4%)	1561 (32%)	1604 (9%)	1653 (35%)
Zn^2+^: GnRH [6-D-Phe] 20_1	1415 (20%) 1447 (5%)	—	1560 (40%)	—	1653 (15%)

### Binding of Zn^2+^ to GnRH [6-D-Phe]

The LHRH interactions with Zn^2+^, Ni^2+^ and Cu^2+^ were studied in the 1980s^17,20^. The results of the ^1^H-NMR spectroscopy favored a view that the coordination between Zn^2+^ and LHRH takes place at the carbonyl oxygen of the His-Trp peptide bond^20^. In addition, the imidazole of the His residue coordinates to the Zn^2+^ ligand. This coordination to Zn^2+^ happens in a fast proton exchange. The changes of the chemical shift in the aromatic part of the ^1^H-NMR in correlation to the added molar concentration of Zn^2+^ can be traced back to two distinct occurrences. The first one involves the already mentioned His and Trp residue interaction with Zn^2+^ (Supplementary Data and Information Fig. 1). The second one can be due to a stronger peptide-peptide interaction^37,38^. Using the observed change in the chemical shift (Δ*δ*), we fitted the data to a hyperbolic function with the following equation:

$${\rm{\Delta }}\delta =\frac{{\rm{\Delta }}{\delta }_{max}\ast Z{n}^{2+}}{{K}_{d}+\,Z{n}^{2+}}$$

**Figure 4:**
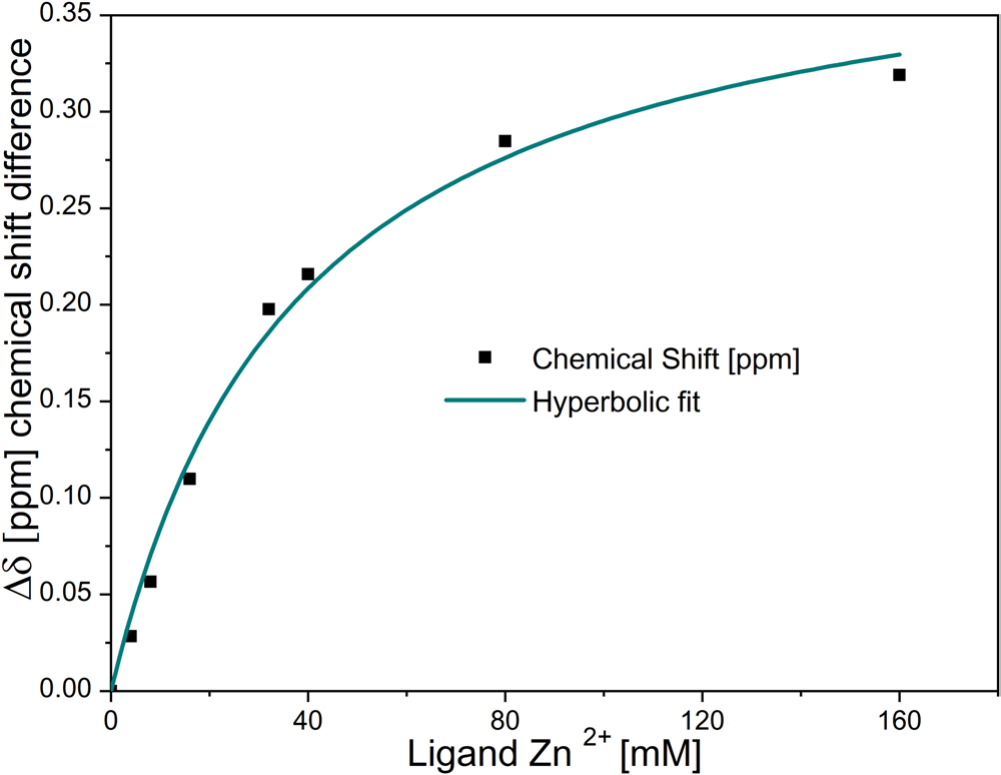
NMR shift in solution-state NMR spectra vs. Zn^2+^ concentration for the determination of the dissociation constant K_d_ of the Zn^2+^: GnRH [6-D-Phe] 1:1 stoichiometry.

This allowed us to determine the dissociation constants (K_d_) of the Zn^2+^: GnRH [6-D-Phe] assembly, assuming a 1:1 stoichiometry. The calculated value of the dissociation constant K_d_ (38.4 ± 4.8 mM^1^) indicated a weak interaction between Zn^2+^ and GnRH [6-D-Phe]. The Zn^2+^ ligand concentration for the interaction with GnRH [6-D-Phe] was in the range of 4–160 mM (Fig. 4).

### Zn^2+^: GnRH [6-D-Phe] assembly: nanostructures and fibrils

**Figure 5:**
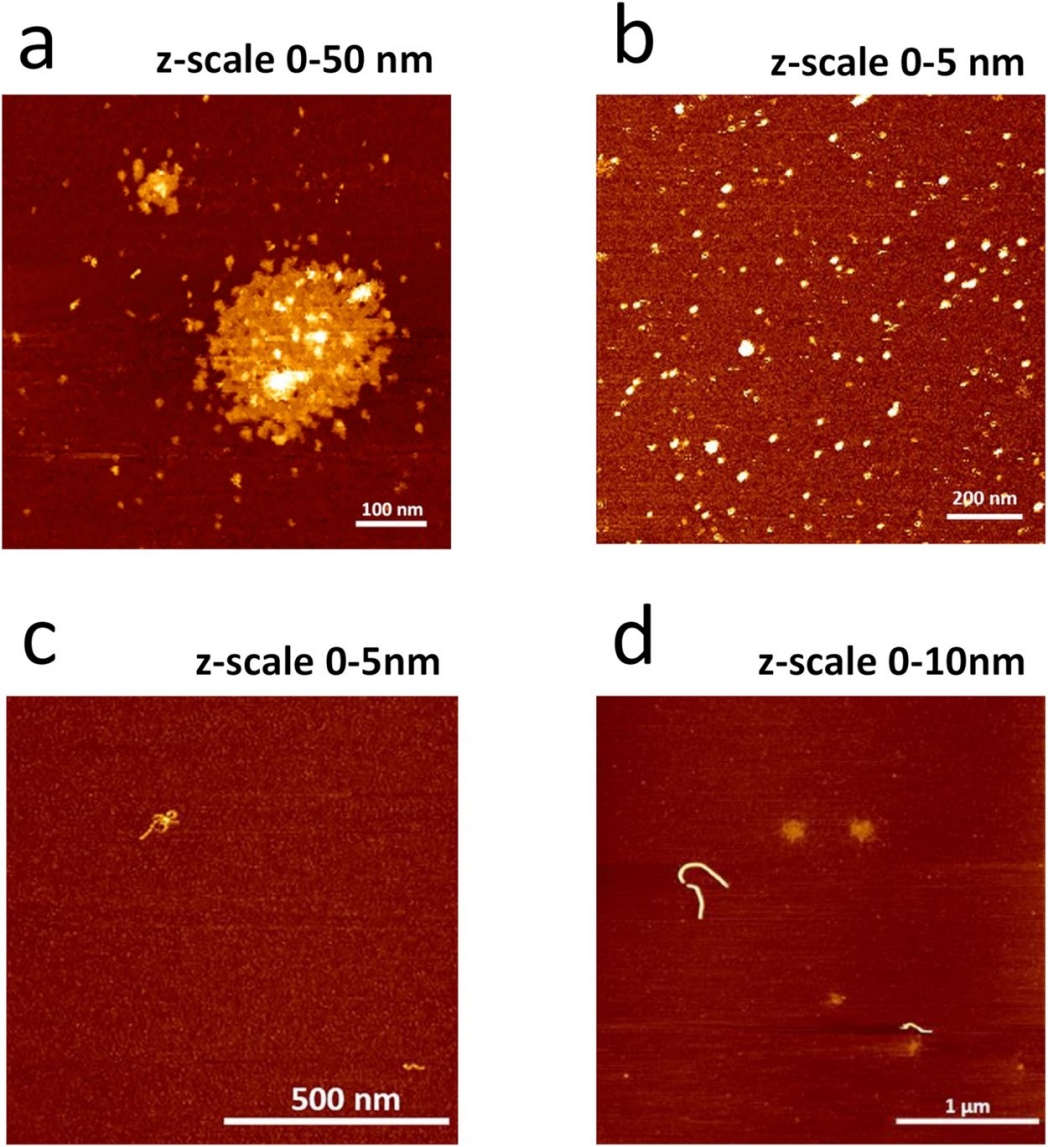
AFM image of the Zn^2+^: GnRH [6-D-Phe] 10:1 complex. (**a**,**b**) Oligomers after preparation with tapping mode in Tris buffer solution (**c**,**d**) fibrils with tapping mode in air (z-scale indicates the average size of the formed oligomers and fibrils).

In order to visualize the formation process of the assembly, we took atomic force microscopy (AFM) images in solution and in dry state at the highest precipitation efficiency, Zn^2+^: GnRH [6-D-Phe] 10:1. The observed fast proton exchange and interaction in the ^1^H-NMR spectra required the use of a D_2_O: DMSO-d6 (80:20) solvent in order to capture the images. In solution, nanostructures with a diameter of up to 5 nm in the presence of Zn^2+^ were recorded (Fig. 5a,b; Supplementary Data and Information Fig. 3). The assembly process appeared to be dynamic and going through different stages: immediately after preparation large circular aggregates formed (Fig. 5a), which resulted after 48 h in 5 nm nanostructures (Fig. 5b). The smaller size oligomers might be due to solvation of peptide chains from the preliminary formed large aggregate back into solution. In dry state, Zn^2+^: GnRH [6-D-Phe] small fibrils with a diameter of up to 10 nm were observed (Fig. 5c,d).

The effect of increasing Zn^2+^ ion concentration on GnRH [6-D-Phe] fibrillization was further studied with Thioflavin T (ThT) assay. ThT is a benzothiazole dye used to visualize and quantify misfolded protein fibrils or amyloid both *in vitro* and *in vivo*^39^. The assay could show the formation of secondary structures with increasing Zn^2+^ concentration. Above a 10:1 Zn^2+^: GnRH [6-D-Phe] ratio, the obtained fluorescence values decreased. Pure GnRH [6-D-Phe] and aggregated Aβ42 were included and served as negative and positive control, respectively (Table 2).

**Table 2:** ThT fluorescence recorded over 24 h of the Zn^2+^: GnRH [6-D-Phe] assemblies in comparison to GnRH [6-D-Phe] and Aβ 42.

	ThT Fluorescence [a.u.] ± SD				
	0 h	1 h	3 h	5 h	24 h
GnRH [6-D-Phe]	**1.1** ± 5.6E-03	**1.2** ± 2.8E-03	**1.2** ± 1.8E-03	**1.1** ± 4.6E-03	**1.1** ± 2.1E-03
2:1	**5.7** ± 8.7E-03	**5.6** ± 2.5E-02	**5.3** ± 1.6E-02	**6.4** ± 5.9E-02	**5.8** ± 1.6E-02
4:1	**7.9** ± 1.3E-02	**6.3** ± 2.0E-02	**7.3** ± 9.2E-03	**5.6** ± 3.4E-02	**5.8** ± 1.7E-02
10:1	**2.5** ± 6.3E-03	**2.8** ± 4.1E-03	**3.3** ± 3.9E-02	**4.9** ± 4.1E-02	**2.4** ± 8.6E-03
20:1	**1.2** ± 3.1E-03	**1.0** ± 5.2E-03	**1.2** ± 7.7E-03	**1.1** ± 9.4E-03	**1.0** ± 7.4E-03
Aβ 42	**5.1** ± 1.8E-02	**3.9** ± 1.1E-02	**5.9** ± 1.2E-01	**4.7** ± 1.9E-01	**4.7** ± 1.9E-01

### Molecular dynamics simulation of Zn^2+^: GnRH [6-D-Phe] 10:1 assembly

**Figure 6:**
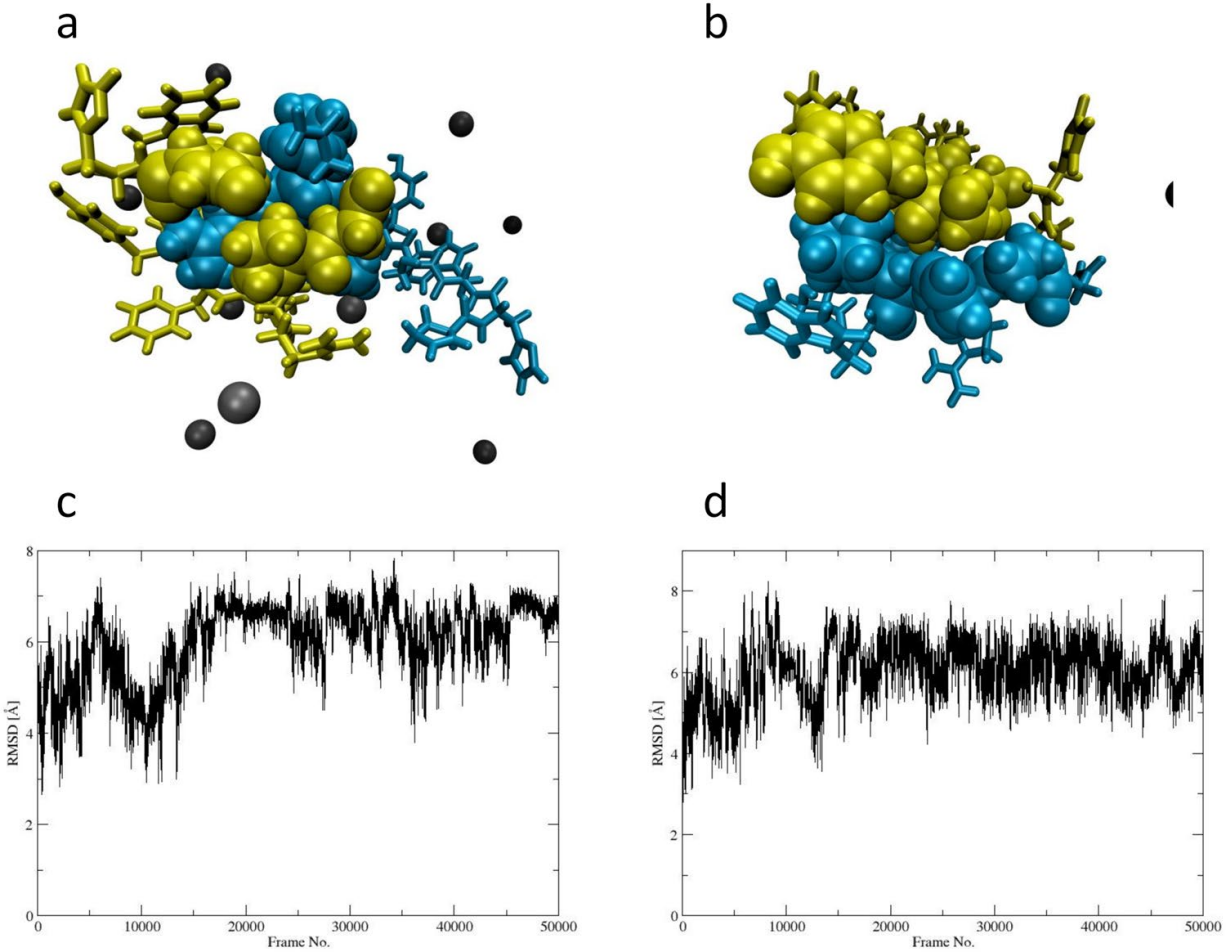
MD simulation. (**a**,**b**) Dimer structures of GnRH [6-D-Phe]-1 in blue, GnRH [6-D-Phe]-2 in yellow, Zn^2+^ in black, interfacial residues pictured as VdW spheres (**c**,**d**) RMSD value peptide 1 and RMSD value peptide 2.

Using molecular dynamics (MD) simulations, we further explored the interaction between Zn^2+^ and GnRH [6-D-Phe] in the 10:1 ratio. Analysis of the trajectory with the linear interaction energy method showed three dimerization events driven by VdW and electrostatic interactions in the Zn^2+^: GnRH [6-D-Phe] 10:1 (Supplementary Data and Information Fig. 4). These interactions could further confirm the observed weak coordination of Zn^2+^ to GnRH [6-D-Phe] in the ^1^H-NMR spectra^40,41^. In the first dimerization event, the Arg8 residue of peptide 1 was engulfed by the second peptide. The dimer’s interface consists of Leu7, Arg8 and Pro9 of peptide 1 and pGlu1, Ser4, Pro9, Gly-NH_2_10 of peptide 2 (Fig. 6a). The second and third dimerization events present a flatter interface, which is composed of residues pGlu1, His2 Tyr5 D-Phe6 Pro9 of peptide 1 and Trp3 Ser4 Tyr5 of peptide 2 (Fig. 6b). During the first dimerization event there was a pronounced conformational change of peptide 1, reflected from the RMSD values (Fig. 6c,d). A cluster analysis showed that the dimer formed in the first dimerization event (at around 10000 frames) is the most prominent species in the course of the simulation. MD simulations showed that the dimer formation is furthermore driven by hydrogen bond formation between the peptide residues (Supplementary Data and Information Fig. 5).

### Self-assembled Zn^2+^: GnRH [6-D-Phe] 10:1 as platform for sustained GnRH [6-D-Phe] delivery in the form of oil depot formulation

**Figure 7:**
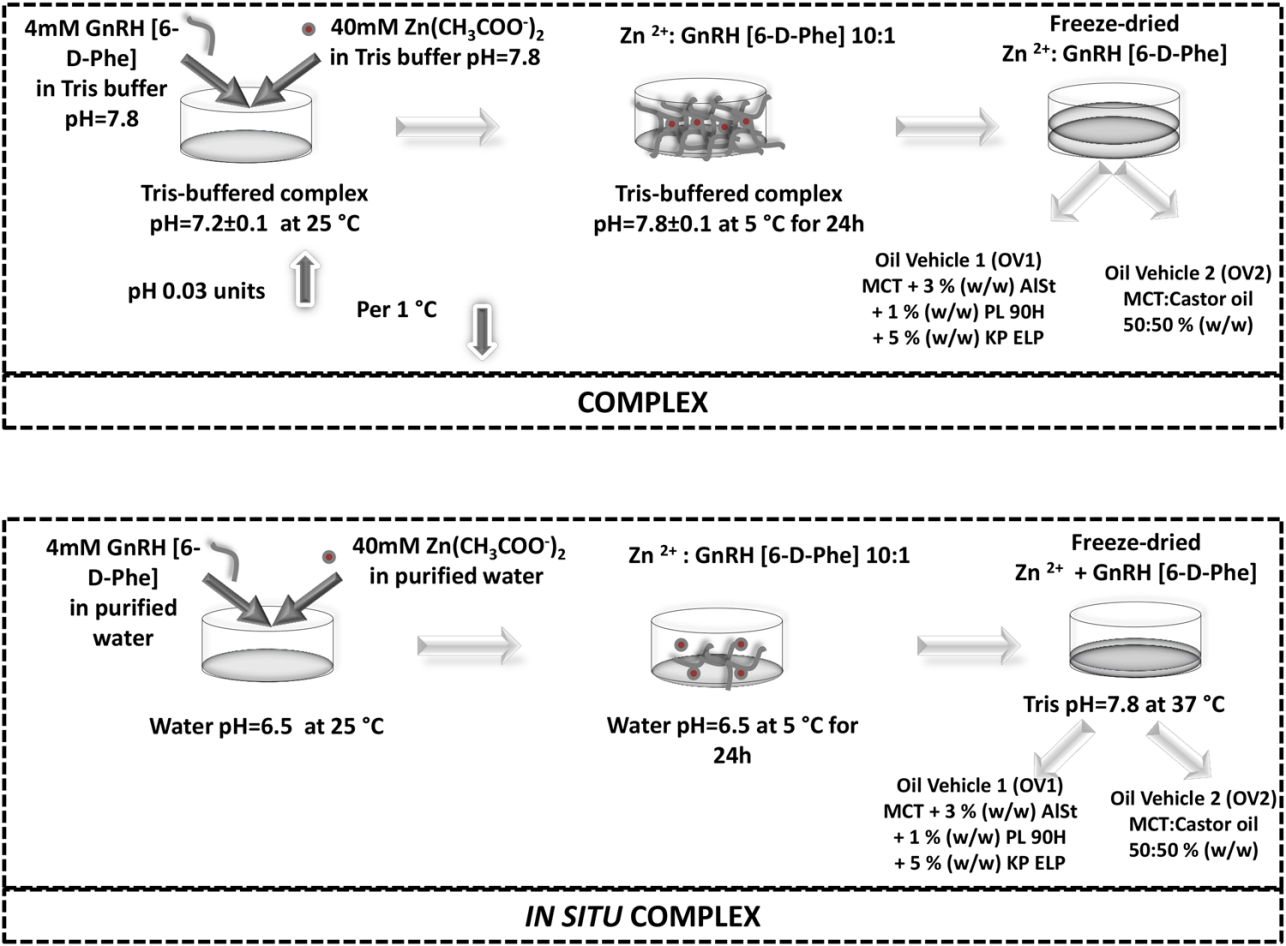
Zn^2+^: GnRH [6-D-Phe] complex and oil depot suspension preparation.

In order to generate a GnRH-depot system two new approaches were evaluated. In the first setup, the nanostructure/fibril formation of GnRH [6-D-Phe] in the presence of Zn^2+^ was directly performed in the freezing phase of a lyophilization process. The temperature-dependent pH switch of the Tris buffer system led to the Zn^2+^ induced self-assembly of GnRH [6-D-Phe], simultaneously stabilized in a 3D freeze-dried matrix (Fig. 7). The complex was then incorporated in medium chain triglycerides (MCT) +3% (w/w) aluminium distearate (AlSt) +1% (w/w) phospholipon 90 H (PL 90 H) +5% (w/w) kolliphor ELP (KP ELP) or in castor oil: MCT 50:50% (w/w) oil vehicle. For the second *in situ* method, the Tris buffer salt was added to the oil vehicle separately from GnRH [6-D-Phe], enabling a possible precipitation and fibrils formations as dissolution of both suspended water soluble compounds takes place after *in vivo* application. Both Zn^2+^: GnRH [6-D-Phe] complexes were characterized and evaluated by an *in vitro* release study.

**Figure 8:**
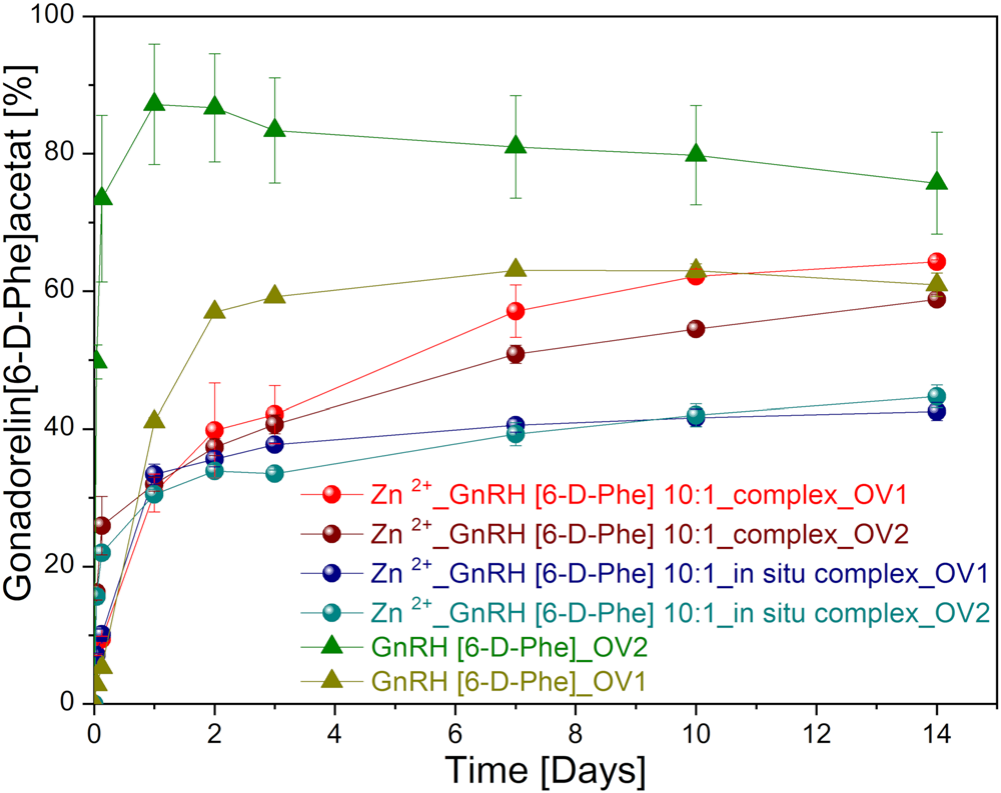
*In vitro* release profiles Zn^2+^: GnRH [6-D-Phe] and GnRH [6-D-Phe] oil depot formulations.

The *in vitro* release of GnRH [6-D-Phe] from the *in situ* complex with Zn^2+^ in the oil vehicle OV1: MCT +3% (w/w) AlSt +1% (w/w) PL 90 H +5% (w/w) KP ELP or OV2: castor oil: MCT 50:50% (w/w) reached a maximum of 40% after 2–3 days (Fig. 8). In contrast, the preformed complex formulations in OV1 and OV2 showed slow and continuous *in vitro* release profiles over 14 days with a minor burst effect. The release from the reference formulation based on pure GnRH [6-D-Phe] in castor oil: MCT 50:50% (w/w) was fast and almost complete within 24 h. In contrast the release was more sustained over 4 days from MCT +3% (w/w) AlSt +1% (w/w) PL 90 H +5% (w/w) KP ELP.

**Figure 9:**
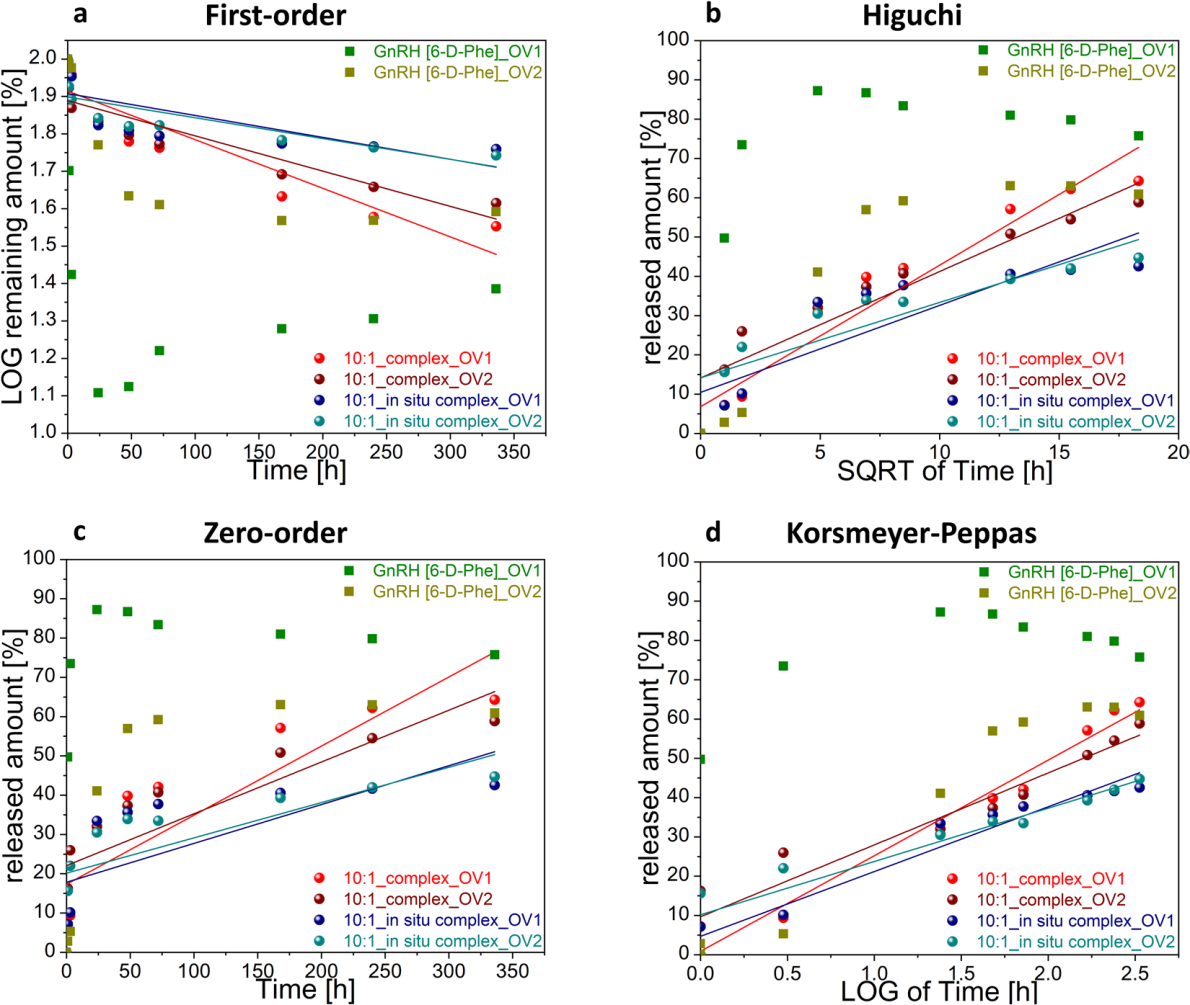
Linear fit of *in vitro* release kinetic values of GnRH [6-D-Phe] from selected Zn^2+^: GnRH [6-D-Phe] and GnRH [6-D-Phe] oil depot formulations. (**a**) ^1^First-order equation, log Q = Log Q_0_ −kt/2.303; (**b**) ^2^Higuchi equation, Q = kt½; (**c**) ^3^Zero-order equation, Q = Q + kt; (**d**) ^4^Korsmeyer-Peppas equation, Q_t_/Q_µ_ = kt^n^.

In order to determine the release kinetics from the Zn^2+^: GnRH [6-D-Phe] formulations, the data was fitted to first-order, Higuchi, zero-order and Korsemeyer-Peppas models^42^. The formulations clearly did not follow zero order release kinetics (Fig. 9). The model that best fitted the release data was evaluated by correlation coefficient (R^2^) (Table 3). The release profile of the preformed complex formulation in OV1 could be best explained by Higuchi model, as the plot showed linearity with R^2^ value of 0.9416. The Korsmeyer-Peppas plots of all formulations showed fair linearity (R^2^ values between 0.95–0.97) with a considerably high slope value (n). This indicated a coupling of diffusion and erosion mechanisms or so called anomalous diffusion^43^.

**Table 3:** *In vitro* release kinetic values of GnRH [6-D-Phe] from selected Zn^2+^: GnRH [6-D-Phe] oil depot formulations.

Formulation	First-order^a^ R^2^	Higuchi^b^ R^2^	Zero-order^c^ R^2^	Korsmeyer-Peppas^d^ R^2^	n
10:1_complex_OV1	0.8574	0.9416	0.7589	0.9784	24.342
10:1_complex_OV2	0.8214	0.8848	0.7087	0.9296	18.314
10:1_*in situ* complex_OV1	0.5505	0.7552	0.5092	0.9533	16.491
10:1_*in situ* complex_OV2	0.6654	0.7891	0.5836	0.8960	13.566
GnRH [6-D-Phe]_OV1	0.5405	0.7460	0.4949	0.9471	27.433
GnRH [6-D-Phe]_OV2	0.0721	0.2894	0.1229	0.5010	19.913

^a^First-order equation, log Q = Log Q_0_ − kt/2.303; ^b^Higuchi equation, Q = kt½; ^c^Zero-order equation, Q = Q + kt; ^d^Korsmeyer-Peppas equation, Q_t_/Q_µ_ = kt^n^.

Compared to the reference formulations without Zn^2+^, the release profiles of the complex and *in situ* complex formulations were not influenced by the oil matrix. Thus the interaction of Zn^2+^ with GnRH [6-D-Phe] in a preformed lyophilized complex could accomplish a superior controlled release of GnRH [6-D-Phe] with minor burst effect. It represents a simple and scalable formulation approach.

## Conclusion

As illustrated above a short synthetic decapeptide, GnRH [6-D-Phe] can form nanostructures and fibrils *in vitro* through the addition of Zn^2+^ ions. The aggregation tendency seems to depend on Zn^2+^ concentration, reaching a maximum efficiency of almost 100% at a 10:1 Zn^2+^: GnRH [6-D-Phe] ratio. The oligomerization and mature fibrils formation of GnRH [6-D-Phe] are Zn^2+^ induced. GnRH [6-D-Phe] fibrils form in a Tris-buffered pH 7.8–8.2 environment in a controlled manner through temperature reduction and a pH shift. The *in vitro* release of the GnRH [6-D-Phe] peptide from the freeze-dried assembly was sustained and continuous over 14 days and can be viewed as a promising platform for the controlled delivery of peptides.

Recent articles have highlighted the numerous fields of application of similar self-assembled peptide –based nanostructures^44–46^. The further investigation of the GnRH [6-D-Phe]: Zn^2+^ self-assembling/oligomerization process is necessary as to evaluate to what extent it can find application in other research fields.

## Materials and Methods

Gonadorelin [6-D-Phe] acetate peptide (pE1–H2–W3–S4–Y5–(D) F6–L7–R8–P9–G10–NH2) or(pGlu-His-Trp-Ser-Tyr-D-Phe-Leu-Arg-Pro-Gly-NH2) was provided by BFC Biopept-Feinchemie aslyophilized powder and acetate salt (purity 99.76%, water content 6.73% and acetate peptide ratio (MW/MW) 1.8). Amyloid β-Protein (1-42) (H-Asp-Ala-Glu-Phe-Arg-His-Asp-Ser-Gly-Tyr-Glu-Val-His-His-Gln-Lys-Leu-Val-Phe-Phe-Ala-Glu-Asp-Val-Gly-Ser-Asn-Lys-Gly-Ala-Ile-Ile-Gly-Leu-Met-Val-Gly-Gly-Val-Val-Ile-Ala-OH) was provided by Bachem Holding AG as lyophilizied powder. Gelling agents: Aluminiumdistearte (Alugel 30 ®HEP), Baerlocher, D-Unterschleissheim); Wetting agents: phospholipon 90 H (Sigma Aldrich, D- Taufkirchen); Resuspendibility enhancer: Kolliphor ELP (BTC Europe, D-Burgbergheim); Complexing agent: Zinc acetate dihydrate (Zn (CH_3_COO^−^)_2_ Merck KGaA, D-Darmstadt); Oil vehicle: miglyol 812 (MCT) (Caesar & Loretz, D-Hilden), castor oil (Gustav Heess, D-Leonberg).

### Formation of the Zn^2+^: GnRH [6-D-Phe] assembly with 0.6 N NaOH

Equal amounts of 4 mM GnRH [6-D-Phe] and 16 mM, 40 mM, 60 mM, 80 mM, 120 mM, 160 mM, 200 mM and 240 mM Zn(CH_3_COO^−^)_2_ aqueous solutions were combined. The pH adjustment was performed using a 0.6 N NaOH under stirring carefully with a dropwise addition along the side wall. The formed precipitate was centrifuged at 12000 × g for 10 min, washed with highly purified water. GnRH [6-D-Phe] in the supernatant as well as in the precipitate was analyzed through a Reversed Phase Chromatography (RP-HPLC) (Supplementary Data and Information Table 1).

### Formation of the Zn^2+^: GnRH [6-D-Phe] assembly in Tris buffer

Equal amounts of 4 mM GnRH [6-D-Phe] and 40 mM, 60 mM, 200 mM Zn(CH_3_COO^−^)_2_ solution in 0.05 M Tris buffer (pH = 7.2, 7.4, 7.6, 7.8, 8.2) were combined. Precipitation was performed at reduced temperature, 5 °C. The formed precipitate was centrifuged at 12000 × g for 10 min, washed with highly purified water. GnRH [6-D-Phe] in the supernatant as well as in the precipitate was analyzed through a RP-HPLC (Supplementary Data and Information Table 2).

### ATR-FTIR

Equal amounts of 4 mM GnRH [6-D-Phe] and 8 mM, 16 mM, 40 mM, 80 mM Zn(CH_3_COO^−^)_2_ solution in a 0.05 M Tris buffer (pH 7.8) were combined. Aliquots of 30 µl of each prepared solution were loaded and the spectra were recorded with a Tensor 27 FTIR spectrometer (Bruker Optics, Ettlingen, Germany) using a Bio-ATR unit at 20 °C. Corresponding blank spectra were subtracted. Spectra were recorded at 4 cm^−1^ resolution and the second derivative spectrum was generated (OPUS, Bruker Optics, Ettlingen, Germany).The spectra data analysis and Fourier Self-Deconvolution (FSD) was performed with Origin 2018 software. FSD was performed using bandwidth of 10 cm^−1^ and noise suppression factor of 0.3. The structure content of the deconvoluted peaks was determined using Gaussian band shapes by an iterative curve fitting.

### NMR Spectroscopy

Solution ^1^H-NMR zinc titration of the peptide in D_2_O: DMSO-d6 (80:20) solvent was performed in a 0.05 M Tris buffer (pH 7.8) at 25 °C on a Bruker Avance III HD 800 MHz and at a reduced temperature 5 °C on a Varian NMR System 400 MHz. The binding efficiency of Zn^2+^ to peptide was determined through the NMR analysis of the Zn^2+^: GnRH [6-D-Phe] assemblies. The spectra were evaluated using the MestReNova Software 10.0.2-15465 (Supplementary Data and Information Fig. 1).

### Atomic force microscopy (tapping in Air)

The 10:1 Zn^2+^: GnRH [6-D-Phe] (20 ng/µL; 20 µL volume) was deposed onto poly-L-lysine coated mica for 2 min, followed by gentle rinsing with water and dried in a N_2_-gas stream. Coating the mica with (+) charged poly-L-lysine repulsed the (+) charged arginine peptide residues of chains not involved in the dimerization process, allowing better imaging. AFM imaging was performed on a Multimode AFM, equipped with a Nanoscope III controller and a type E scanner. Images were recorded on dried samples, under ambient conditions, and using Silicon cantilevers (Olympus; AC160TS; resonance frequency ≈300 kHz). Typical scans were recorded at 1–3 Hz line frequency, with optimized feedback parameters and at 512 × 512 pixels. For image processing and analysis, Scanning Probe Image Processor (v6.4; Image Metrology) was employed. Image processing involved background correction using global fitting with a third-order polynomial and line-by-line correction through the histogram alignment routine.

### Atomic force microscopy (tapping in Tris buffer solution)

The AFM images were performed using the AFM NanoWizard® 4 from JPK Instruments with SPM software and an integrated Axiovert 200 inverted microscope from Zeiss. As cantilever the qp-BioAC CB3 (Nanosenors; resonance frequency ≈30 kHz) was used. A mica-chip was glued on a microscope slide and 20 µl of the 10:1 Zn^2+^: GnRH [6-D-Phe] was added. After 5 min incubation time, the mica-substrate was washed with Tris buffer. The imaging was performed in 1 ml Tris buffer and with the tapping mode (QI™ Advanced Imaging). The following values have been set: Z-length 50–70 nm; duration 2 ms and pixel size 256 × 256. All images were processed and optimized with Data Processing Software from JPK. The processing involved removing incorrect lines, replacing outlier pixel values with the median value of neighbouring pixels and line-by-line correction with a first-order polynomial fit. A histogram is calculated for each line and only the data between the lower and upper limits is used for fitting the polynomial.

### Thioflavin-T Binding Assay

The fluorescence intensity was measured in multi-well plates in an Agilent Cary Eclipse Fluorescence Spectrophotometer (exc.at 440 nm (slit width 5 nm), em. at 482 nm (slit width 10 nm), and averaging over 60 s). 100 µl of each Zn^2+^: GnRH [6-D-Phe] sample, prepared in 0.05 M Tris buffer pH 7.8 containing 20% DMSO, was vortexed gently and mixed with 200 µL 50 µM ThT solution prepared in the same buffer and stored at 5 °C. Aβ42 lyophilizate was dissolved in 0.05 M Tris pH 7.8 containing 20% DMSO and stored at 5 °C over 24 h for the purpose of forming minimal peptide aggregation. 100 µl of 150 µM Aβ42 and 100 µl of 4 mM GnRH [6-D-Phe] were used as controls and treated the same way as the Zn^2+^: GnRH [6-D-Phe] samples. The fluorescence intensity of all samples was measured over 24 hours.

### MD simulations

Molecular dynamic simulations were performed using the Amber 16 suite^47^. Non-standard residue files were generated using antechamber and the peptide structure was generated with t-leap. Two identical GnRH [6-D-Phe] molecules and 20 zinc ions were placed at random coordinates in a TIP3P water box using 10 Å of padding. Energy minimization was performed with pmemd. MPI with the steepest descent method for 5000 cycles and with the conjugate gradient method at constant volume for the remaining 45000 cycles. The system was then heated from 0 to 300 K and equilibrated for 200 ps with pmemd.cuda. A Langevin thermostat with a collision frequency set to 5 ps^−1^ was used. Pressure was kept constant using isotropic position scaling and a Monte Carlo barostat. The SHAKE algorithm was turned on. After equilibration, a 500 ns production run was performed with the same thermostat and barostat settings as during equilibration. VdW and electrostatic interaction energies were calculated using the LIE method as implemented in cpptraj and plotted with matplotlib^40,41,48^.

### Zn^2+^: GnRH [6-D-Phe] complex preparation

Zn^2+^: GnRH [6-D-Phe] 10:1 preformed complex was prepared from equal amounts of 4 mM GnRH [6-D-Phe] and 40 mM Zn(CH_3_COO^−^)_2_ in 0.05 M Tris buffer pH 7.8. The pH of the resulting mixture at 25 °C was 7.2. The temperature was then reduced to 5 °C and resulted in a pH increase to pH 7.8. The mixture was kept at 5 °C for 24 h and then freeze dried. The *in situ* Zn^2+^: GnRH [6-D-Phe] 10:1 complex was prepared from equal amounts of 4 mM GnRH [6-D-Phe] and 40 mM Zn (CH_3_COO^−^)_2_ in water. The pH of the resulting mixture at 25 °C was 6.5. Similarly to the preformed complex the temperature was reduced to 5 °C. The pH stayed constant. The mixture was kept at the same conditions as the preformed complex and freeze dried. Both complexes were freeze dried using a Christ Epsilon 2–6 D freeze-dryer (Martin Christ Gefriertrocknungsanlagen GmbH, D-Osterode) (150 mTorr; primary drying: 60 h, −20 °C; secondary drying: 18 h, 20 °C) with process and plant control LPC plus SCADA software.

### Zn^2+^: GnRH [6-D-Phe] lyophilisate micronization

The dry- cryogenic micronization of Zn^2+^: GnRH [6-D-Phe] lyophilisate and the separately added tris buffer were performed using a Retsch Cryo Mill (Retsch Technology, Haan, Germany). The grinding process was performed for the duration of 1 min at 25 Hz with a precooling phase of 10 min in liquid nitrogen.

### Zn^2+^: GnRH [6-D-Phe] oil depot preparation

The Zn^2+^: GnRH [6-D-Phe] oil depot formulations preparation was performed under a laminar flow cabinet in a two-step process. The gelling agent Aluminiumdistearte Alugel 30 ®HEP (AlSt) was weighed and suspended in the oil vehicle miglyol 812 (MCT) to a final weight of 95 g (corresponding to 100 mL). The prepared mixture was heated at 174 °C for 2 hours under N_2_. After cooling down to 25 °C the wetting agent, Phospholipon 90 H (PL 90 H), and the resuspendibility enhancer, Kolliphor ELP (KL ELP), were incorporated into the gelled oil matrix and stirred at 160 °C for 1 hour under N_2._ The castor oil: MCT 50:50% (w/w) matrix was heated and agitated under inert atmosphere (N_2_) at 60 °C and then cooled down to room temperature at 25 °C. The cryo-ground Zn^2+^: GnRH [6-D-Phe] lyophilizate was suspended in the prepared oil matrices at ambient temperature of 25 °C using an Ultra-Turrax T-10 basic (IKA Labortechnik, Germany) for 5 minutes at 2000 rpm. 139.8 mg of Tris buffer salt consisting of 2.01 g Trizma HCl and 1.485 g Trizma Base was added to the *in-situ* complex formulation. The 5 mg/mL Zn^2+^: GnRH [6-D-Phe] oil depot suspensions were aliquoted in 20 R glass vials. The prepared suspensions were stored at 2–8 °C.

### GnRH [6-D-Phe] extraction from the complex and oil matrix

In order to establish a suitable extraction method for the determination of the GnRH [6-D-Phe] content in the oil depot suspension the following extraction experiments were performed. The GnRH [6-D-Phe] was extracted from the prepared Zn^2+^: GnRH [6-D-Phe] oil depot suspensions using dichloromethane (DCM), where GnRH [6-D-Phe] is not soluble in combination with HEPES (pH 7.4). HEPES was selected as medium instead of PBS buffer, since phosphate can precipitate the zinc salt from the complex. 2 mL oil depot suspension was weighted into a falcon tube, 4 mL DCM and 6 mL HEPES (pH 7.4) were added. The tube was shaken or vortexed at room temperature (25 °C) and put into an incubated shaker 3031 (GFL, Germany) at 39 °C and 60 rpm for 48 h. The RP-HPLC analysis was performed at 220 nm. A second extraction method was applied: 2 ml of each formulation were pipetted into 6 ml HEPES (pH 7.4). The falcon tubes were placed in an incubated shaker 3031 (GFL, Germany) at 39 °C and 60 rpm for 48 h. The tubes were then centrifuged for 60 minutes at 4000 rpm and 5 °C. 1 ml of the lower aqueous phase was used for RP-HPLC at 220 nm. The second extraction with HEPES (pH 7.4) delivered results of nearly 100% and indicated high extraction efficiency.

### *In vitro* release study

The *in vitro* release study was conducted using VISKING® dialysis tubing, MWCO 12–14 kD, RC, 28 mm (SERVA, Germany). The tubes were filled with 1.5 mL formulation. The release medium was 20 mL HEPES (pH 7.4). HEPES was selected as release medium instead of PBS buffer, since phosphate can precipitate the zinc salt from the complex. The *in vitro* evaluation was performed in duplicates and in an incubated shaker 3031 (GFL, Germany) at 39 °C and 60 rpm. 1 mL sample was used for the RP-HPLC analysis at the following time points: 1 h, 3 h, 5 h, 7 h, 22 h, 25 h, 28 h, 46 h, 52 h, 76 h, 100 h, 172 h, 196 h, 220 h, and 336 h (14 days). The GnRH [6-D-Phe] content in the oil vehicle and in the donor cell was extracted using 2 mL HEPES buffer (pH 7.4). After 48 hours of an equilibration of the peptide between the two phases the emulsion was centrifuged for 60 minutes at 4000 rpm and 5 °C. The quantity of the peptide in the lower aqueous phase was analysed by RP-HPLC at 220 nm.

### Determination of the GnRH [6-D-Phe] (RP-HPLC)

The GnRH [6-D-Phe] content in the formed precipitate/fibrils was analysed by RP-HPLC after dissolution in 0.1% acetic acid, using a LUNA C8 (4.6 × 250 mm; size = 5 µm; Phenomenex, USA) column, with a C8 pre-column (4 × 3 mm; size = 5 µm) at an HPLC Agilent 1100/1200 series (Agilent Technologies, USA) (mobile phase A (water +1 mL/L Trifluoroacetic acid (TFA) (v/v)) and mobile phase B (800 g Acetonitrile +200 g water +1.2 mL TFA), 1.1 mL/min flow, column temperature 40 °C, and autosampler temperature 2–8 °C. The Retention Time (RT) of GnRH [6-D-Phe] was 8.5 ± 1.5 minutes with UV detection at 220 nm.

## Electronic supplementary material

Supplementary Information
