# Supplementary material for: Zn2+-triggered self-assembly of Gonadorelin [6-D-Phe] to produce nanostructures and fibrils
Source: Sci Rep. 2018 Jul 26;8:11280. doi: 10.1038/s41598-018-29529-w (PMC6062538; doi:10.1038/s41598-018-29529-w)
Supplement: Supplementary file 1 — Supplementary Information [file 41598_2018_29529_MOESM1_ESM.pdf]

Zn^{2+}-triggered self-assembly of Gonadorelin [6-D-Phe] to produce nanostructures and fibrils

Yordanka Yordanova[1,*], Willem Vanderlinden[2], Raphael Stoll[3], Daniel Ruediger[4],Andreas Tosstorff[1], Wolfgang Zaremba[5], Gerhard Winter[1], Stefan Zahler[4], Wolfgang Friess[1]

1. Department of Pharmacy, Pharmaceutical Technology & Biopharmaceutics, Ludwig-Maximilians-Universitaet, Butenandtstrasse 5, 81377 Muenchen, Germany
2. Department of Applied Physics and Centre for NanoScience, Ludwig-Maximilian-Universitaet, Amalienstrasse 54, 80799 Muenchen, Germany
3. Department of Chemistry and Biochemistry, Biomolecular NMR Spectroscopy and RUBiospek, Ruhr-Universitaet Bochum, Universitaetsstrasse 150, 44780 Bochum, Germany
4. Department of Pharmacy, Pharmaceutical Biology, Ludwig-Maximilians-Universitaet, Butenandtstrasse 5, 81377 Muenchen, Germany
5. Veyx Pharma GmbH, Scientific Department, Soehreweg 6, 34639 Schwarzenborn, Germany

Correspondence to: Wolfgang Friess, Yordanka Yordanova Correspondence and requests for materials should be addressed to Wolfgang Friess (wolfgang.friess@cup.uni-muenchen.de), Yordanka Yordanova (Email: yordanka.yordanova@cup.uni-muenchen.de)

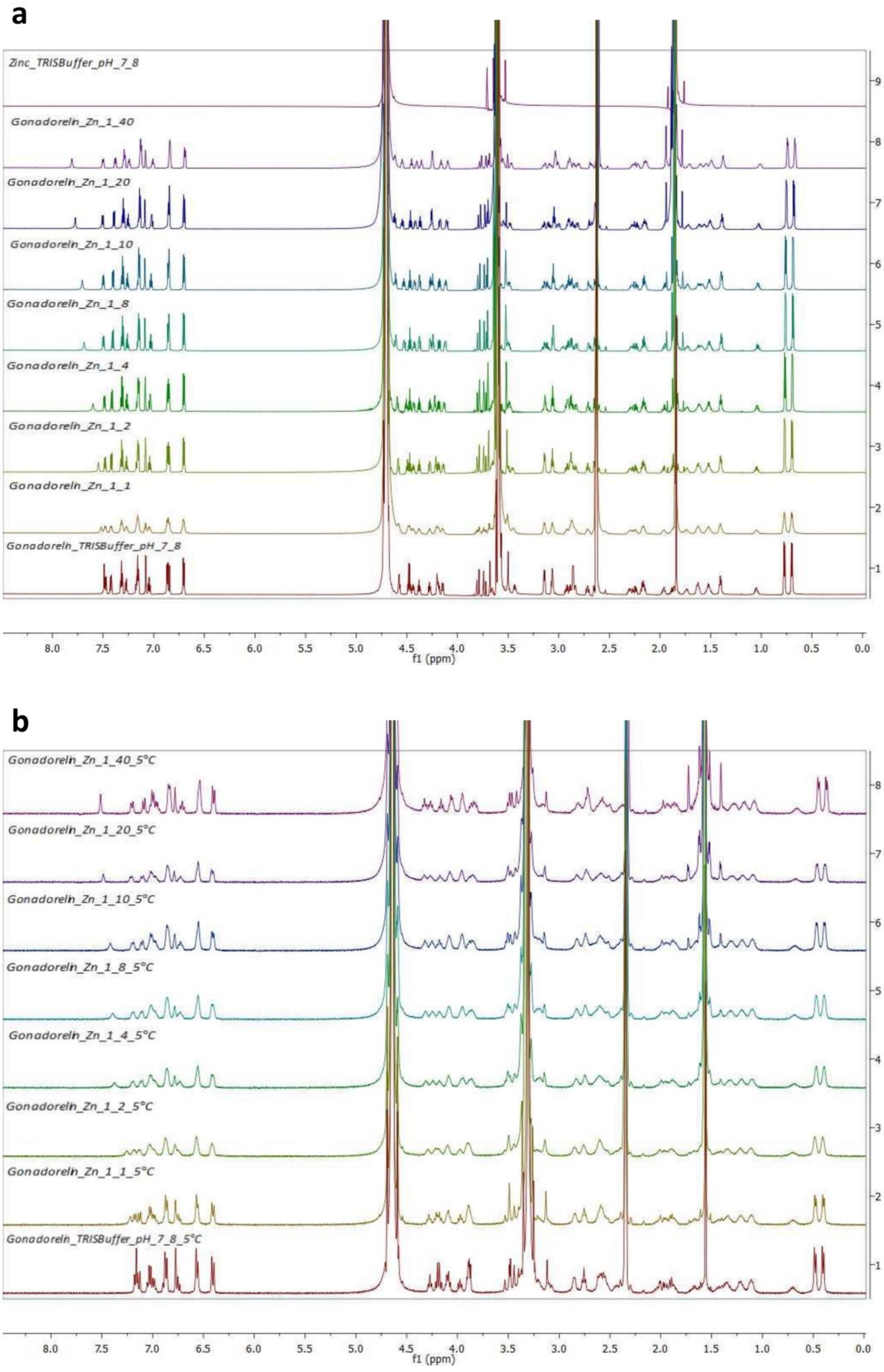

Figure 1| Solution-state NMR Spectra of Zn^{2+}: GnRH [6-D-Phe] assemblies
molar ratio 1:1 , 2:1, 4:1, 8:1,10:1, 20:1, 40:1, GnRH [6-D-Phe] in Tris buffer (pH 7.8) **(a)** at 25 °C **(b)** at 5 °C

1	2	3		4	
Ratio Zn $^{2+}$: GnRH	pH at 25°C	pH adjusted with 0.6 N NaOH	GnRH [6-D-Phe] [%] ± SD	pH adjusted with 0.6 N NaOH	GnRH [6-D-Phe] [%] ± SD
4:1	6.4	7.0	1.2 ± 0.2	8.2	9.8 ± 0.2
10:1	6.4	7.0	1.2 ± 0.2	8.3	95.9 ± 0.1
15:1	6.5	7.8	41.5 ± 0.1	8.3	28.4 ± 0.1
20:1	6.5	7.1	6.8 ± 0.1	8.1	33.9 ± 0.3
30:1	6.6	7.2	15.2 ± 0.2	8.1	44.2 ± 0.1
40:1	6.6	7.3	26.3 ± 0.1	8.2	54.7 ± 0.3
50:1	6.6	7.2	24.9 ± 0.2	8.0	54.9 ± 0.2
60:1	6.6	6.8	13.3 ± 0.1	8.1	61.2 ± 0.3

Table 1| Precipitation of the Zn $^{2+}$: GnRH [6-D-Phe] assembly with 0.6 N NaOH
Column 1 indicates the Zn^{2+}: GnRH molar ratios. **Column 2** represents the pH of the corresponding aqueous Zn^{2+}: GnRH at 25°C. **Column 3** represents the adjusted pH at ~ 7.0 with 0.6N NaOH and corresponding % precipitated peptide. **Column 4** represents the adjusted pH at ~ 8.0 with 0.6N NaOH and corresponding % precipitated peptide. The titration with NaOH did not offer enough precision to adjust the pH value of the solutions in the range of pH=7.2- 8.2 at 0.2 pH units step

1	2	3	4	5	2	3	4	5	2	3	4	5
pH Tris Buffer	Ratio Zn2+: GnRH	pH 25°C	pH 5°C	GnRH [6-D-Phe] at 5° C [%] ± SD	Ratio Zn2+: GnRH	pH 25°C	pH 5°C	GnRH [6-D-Phe] at 5° C [%] ± SD	Ratio Zn2+: GnRH	pH 25°C	pH 5°C	GnRH [6-D-Phe] at 5° C [%] ± SD
7.2		6.7	7.3	36.2 ±0.1		6.7	7.3	no precipitation		6.3	6.9	no precipitation
7.4		6.9	7.5	53.6 ±0.1		6.8	7.4	40.5 ±0.1		6.4	7.0	no precipitation
7.6	10:1	7.1	7.7	59.9 ±0.1	15:1	7.0	7.6	48.8 ±0.1	50:1	6.6	7.2	no precipitation
7.8		7.3	7.9	99.9 ±0.2		7.1	7.7	50.5 ±0.1		6.8	7.4	12.2 ±0.1
8.2		7.5	8.1	45.7 ±0.1		7.2	7.8	13.9 ±0.1		6.7	7.3	16.2 ±0.1

Table 2| Precipitation of the Zn $^{2+}$: GnRH [6-D-Phe] assembly in Tris buffer (pH 7.8) at 5 °C
Column 1 represents the pH of the used Tris-buffer. **Column 2** indicates the Zn^{2+}: GnRH molar ratio. **Column 3** represents the pH of Zn $^{2+}$: GnRH [6-D-Phe] assembly in the corresponding Tris-buffer at 25°C. **Column 4** represents the pH of Zn $^{2+}$: GnRH [6-D-Phe] assembly in the corresponding Tris-buffer at 5°C. **Column 5** represents the corresponding % precipitated peptide after reduction of 25 °C to 5°C over 24h

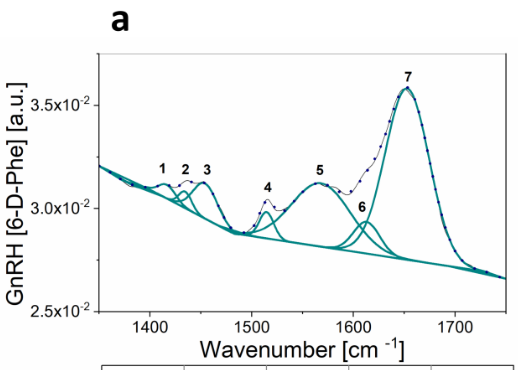

Index	Center	FWHM	Height	Area
1	1416.74	19.96	6.64E-0	1.71
2	1434.73	14.92	8.01E-0	1.54
3	1454.28	28.35	1.70E-0	6.24
4	1514.58	16.89	1.28E-0	2.78
5	1568.23	67.85	3.07E-0	26.94
6	1612.96	30.58	1.54E-0	6.07
7	1652.86	50.98	8.31E-0	54.72

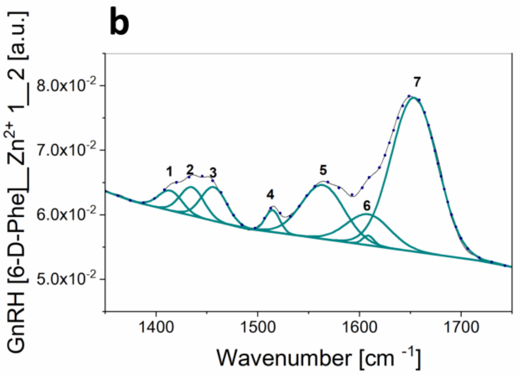

Index	Center	FWHM	Height	Area
1	1414.00	27.12	3.07E-0	3.53
2	1435.36	27.34	4.41E-0	5.11
3	1456.98	29.45	5.25E-0	6.55
4	1514.51	16.68	3.37E-0	2.38
5	1563.86	49.33	8.35E-0	17.46
6	1608.72	49.95	4.79E-0	10.15
6	1608.72	13.33	1.49E-0	0.84
7	1653.84	53.38	2.38E-0	53.98

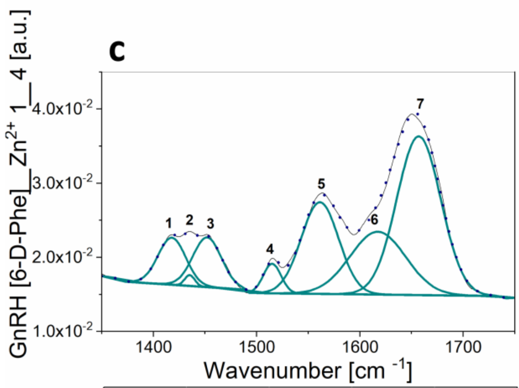

Index	Center	FWHM	Height	Area
1	1417.99	31.42	6.34E-0	7.52
2	1435.26	11.28	1.46E-0	0.62
3	1452.18	32.62	6.70E-0	8.25
4	1515.06	20.21	3.98E-0	3.03
5	1561.29	42.77	1.24E-0	19.93
6	1617.46	63.33	8.47E-0	20.23
7	1657.03	50.06	2.14E-0	40.42

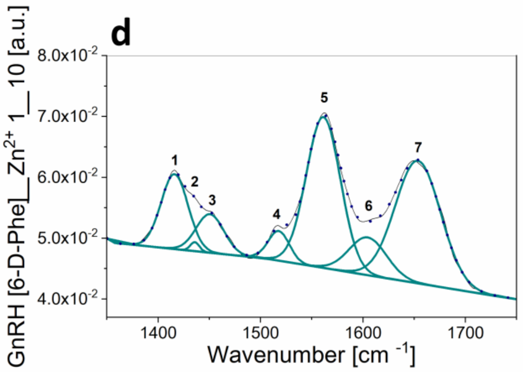

Index	Center	FWHM	Height	Area
1	1416.08	30.86	1.23E-0	12.65
2	1435.92	10.30	1.47E-0	0.50
3	1450.71	31.73	6.33E-0	6.70
4	1517.83	24.16	4.86E-0	3.92
5	1561.35	39.17	2.48E-0	32.38
6	1604.70	42.78	6.20E-0	8.84
7	1653.68	52.12	2.01E-0	35.00

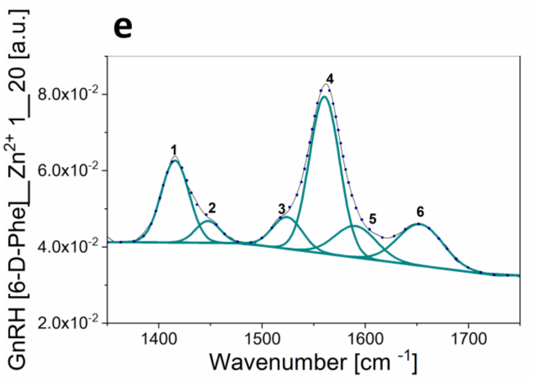

Index	Center	FWHM	Height	Area
1	1415.62	31.65	2.15E-0	19.56
2	1447.52	28.24	5.71E-0	4.64
3	1524.39	34.14	8.31E-0	8.16
4	1560.47	34.26	4.11E-0	40.51
5	1590.54	47.04	8.26E-0	11.17
6	1653.32	51.00	1.09E-0	15.96

Figure 2| Deconvoluted FT-IR absorbance spectra
(a) GnRH [6-D-Phe] and **(b)** 2:1**;** **(c)** 4:1; **(d)** 10:1 **;** **(e)** 20:1 Zn^{2+}: GnRH [6-D-Phe] assemblies

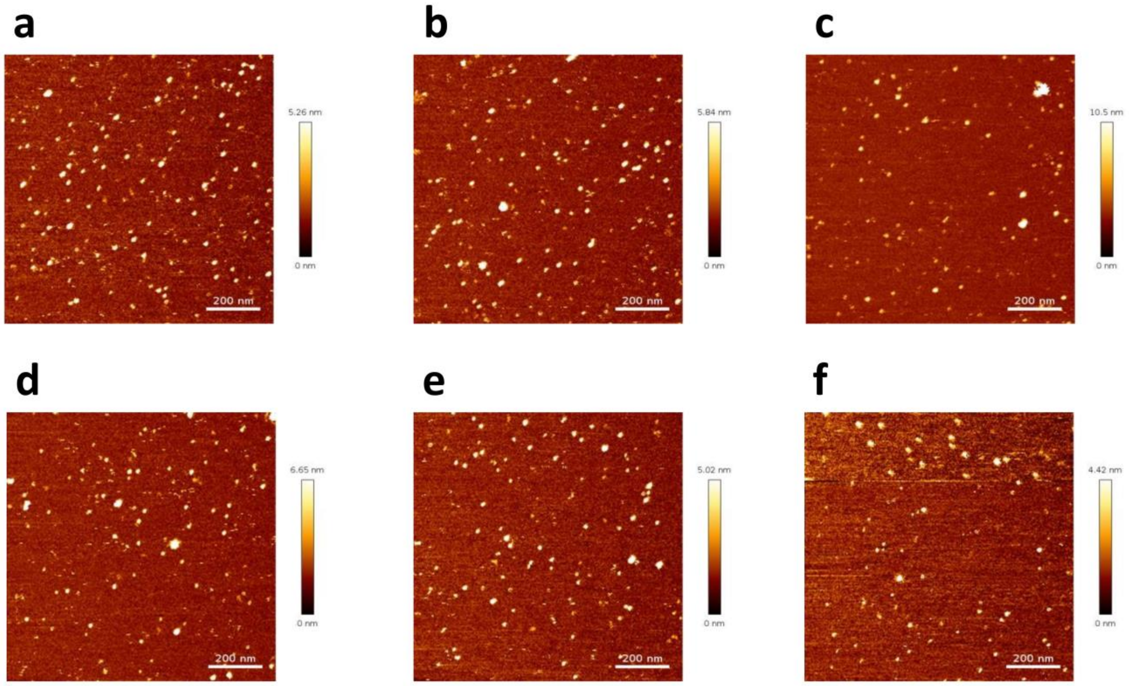

Figure 3| AFM image of the Zn $^{2+}$: GnRH [6-D-Phe]
(a)-(e) tapping mode Tris buffer solution with dilution 1:1000 after 48h

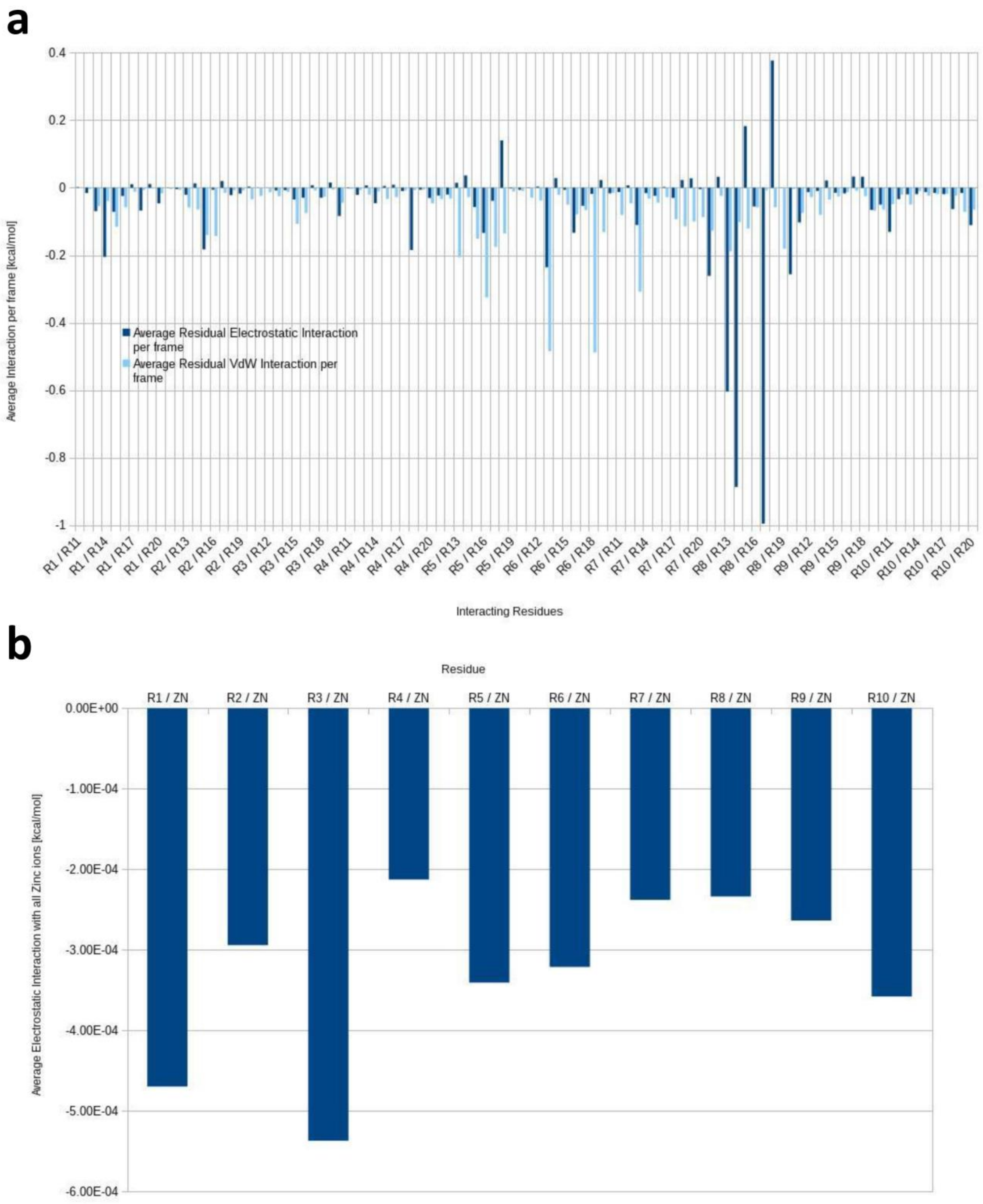

Figure 4| Average linear interaction energy

(a) VdW and electrostatic of R1-R10 amino acid residue of the first peptide chain and R11-R20 amino acid residue of the second peptide chain **(b)**electrostatic of all amino acid residue with Zn $^{2+}$

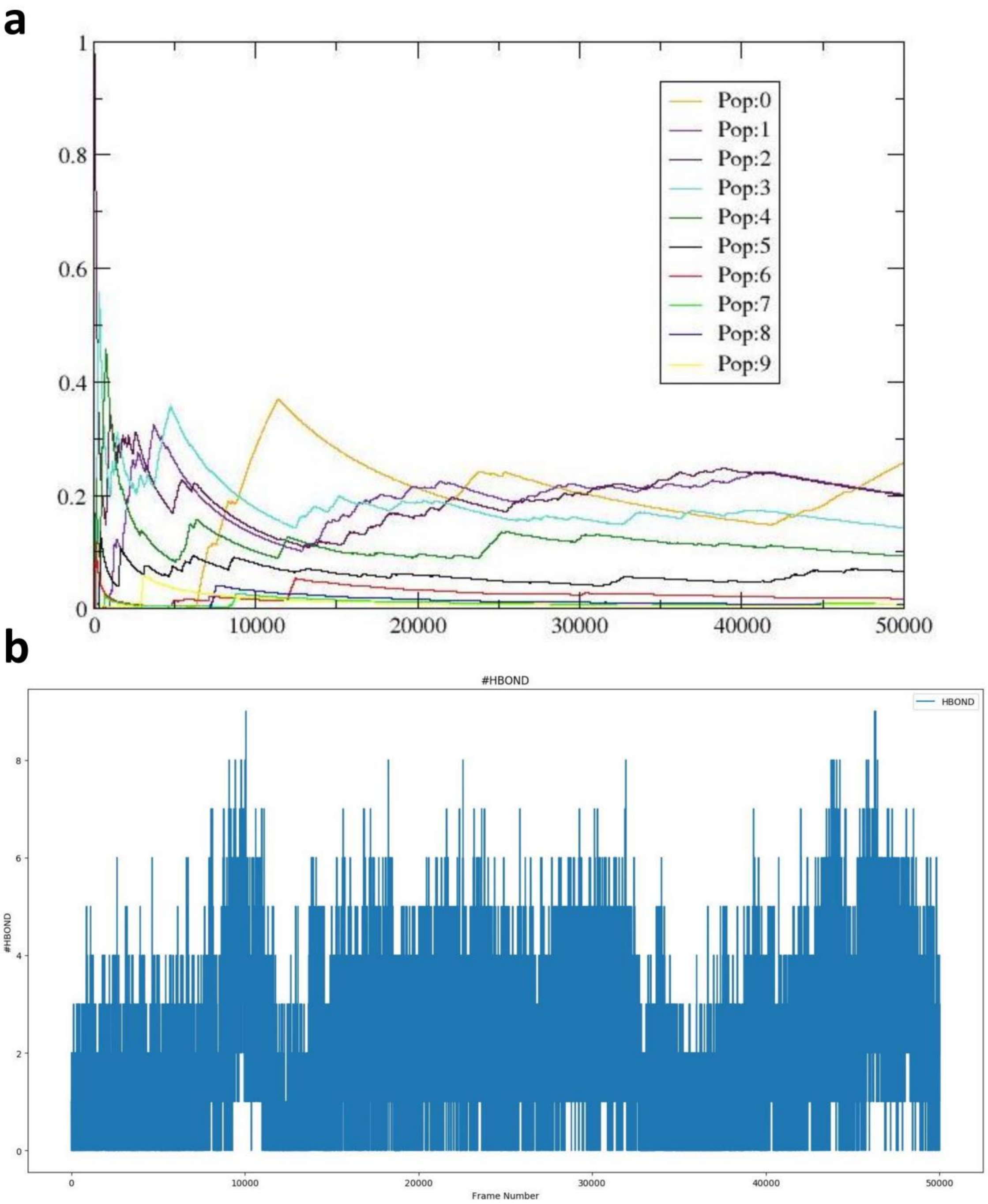

Figure 5| Peptide dimerization
(a) Cluster analysis of the formed dimer **(b)** Total number of hydrogen bonds formed between peptide residues during a 500 ns MD simulation.

Figures and Tables

Figure 1| Solution-state NMR Spectra of Zn $^{2+}$: GnRH [6-D-Phe] assemblies.. 2
Figure 2| Deconvoluted FT-IR absorbance spectra.. 4
Figure 3| AFM image of the Zn $^{2+}$: GnRH [6-D-Phe] 5
Figure 4| Average linear interaction energy.. 6
Figure 5| Peptide dimerization 7

Table 1| Precipitation of the Zn $^{2+}$: GnRH [6-D-Phe] assembly with 0.6 N NaOH 3
Table 2| Precipitation of the Zn $^{2+}$: GnRH [6-D-Phe] assembly in Tris buffer (pH 7.8) at 5 °C 3
